# Supplementary material for: Total Antioxidant Capacity in Obese and Non-Obese Subjects and Its Association with Anthropo-Metabolic Markers: Systematic Review and Meta-Analysis
Source: Antioxidants (Basel). 2023 Jul 28;12(8):1512. doi: 10.3390/antiox12081512 (PMC10451178; doi:10.3390/antiox12081512)
Supplement: Supplementary file 1 [file antioxidants-12-01512-s001.zip › antioxidants-2516633-supplementary.pdf]

Supplementary Table S1. Query

**Search: (obesity) AND (total antioxidant capacity) Sort by: Most Recent**<br>("obeses"[All Fields] OR "obesity"[MeSH Terms] OR "obesity"[All Fields] OR "obese"[All Fields] OR "obesities"[All Fields] OR "obesity s"[All Fields]) AND (("total"[All Fields] OR "totaled"[All Fields] OR "totaling"[All Fields] OR "totalled"[All Fields] OR "totalling"[All Fields] OR "totals"[All Fields]) AND ("antioxidant s"[All Fields] OR "antioxidants"[Pharmacological Action] OR "antioxidants"[MeSH Terms] OR "antioxidants"[All Fields] OR "antioxidant"[All Fields] OR "antioxidating"[All Fields] OR "antioxidation"[All Fields] OR "antioxidative"[All Fields] OR "antioxidatively"[All Fields] OR "antioxidatives"[All Fields] OR "antioxidizing"[All Fields]) AND ("capacities"[All Fields] OR "capacity"[All Fields]))
**Translations**<br>**obesity:** "obeses"[All Fields] OR "obesity"[MeSH Terms] OR "obesity"[All Fields] OR "obese"[All Fields] OR "obesities"[All Fields] OR "obesity's"[All Fields]<br>**total:** "total"[All Fields] OR "totaled"[All Fields] OR "totaling"[All Fields] OR "totalled"[All Fields] OR "totalling"[All Fields] OR "totals"[All Fields]<br>**antioxidant:** "antioxidant's"[All Fields] OR "antioxidants"[Pharmacological Action] OR "antioxidants"[MeSH Terms] OR "antioxidants"[All Fields] OR "antioxidant"[All Fields] OR "antioxidating"[All Fields] OR "antioxidation"[All Fields] OR "antioxidative"[All Fields] OR "antioxidatively"[All Fields] OR "antioxidatives"[All Fields] OR "antioxidizing"[All Fields]<br>**capacity:** "capacities"[All Fields] OR "capacity"[All Fields]

Supplementary Table S2. Altitude where study was conducted, criteria employed for obesity classification, methods used for determining serum TAC, other oxidative stress related parameters measured and their correlations with metabolic and anthropometric parameters.

**Author, year**	**Country**	**Altitude (m)**	**Criteria for NW and obesity (Kg/m2)**	**Methods used for determining serum TAC**	**Other oxidative stress related parameters**	**Correlation of other oxidative stress parameters with metabolic and anthropometric parameters**
Amirkhizi (2010)	Iran	1.768	WHO criteria<br><br>NW: BMI <25.0 kg/m2<br><br>Obesity: BMI ≥ 30 kg/m2	Colorimetric assay using 2,2′-Azino-di-[3-ethyl-benzthiazoline sulphonate] (ABTS) (Miller NJ)	Plasma MDA concentra- tion was assayed by measurement of thiobarbituric acid reactive substances (TBARS) according to Satoh method<br>NW: 1.96<br>OB: 3.25	Plasma **MDA** was significantly related to Weight (0.185, $p<0.01$), BMI (0.484, $p<0.0001$), Waist circumference (0.582, $p<0.0001$), and waist-to-hip ratio (0.474, $p<0.0001$).
Asghari (2021)	Iran	482	WHO criteria<br><br>NW: BMI <25.0 kg/m2<br><br>Obesity: BMI ≥ 30 kg/m2	Colorimetric assay using 2, 20-Azino-di-[3-ethylbenzthiazoline sulphonate] (ABTS) (Erel)	-Superoxide dismutase (SOD): NW:934.1; OB: 1,129.86<br>-Glutathione peroxidase (GPx): NW: 40.7; OB: 44.1<br>-Catalase (CAT): NW: 234; OB:216.6<br>-Paraoxonase-1 (PON1): NW: 60.1; OB: 39.0<br>-Arylesterase (AREase) activity: NW: 225; OB: 182.6<br>-MDA (TBARS): NW: 1.5; OB: 1.8<br>-Total oxidant status (TOS): NW: 9.5; OB:	No correlations were made between the parameters: SOD, GPx, CAT, PON1, AREase and **TOS**

					10.9	

Aslan (2017)	Turkey	1.675	WHO criteria<br><br>NW: BMI <25.0 kg/m2<br><br>Obesity: BMI ≥ 30 kg/m2	Colorimetric using Fenton-type reaction 2,29-azino-bis (3-ethylbenz-thiazoline-6-sulfonic acid) radical cation by antioxidants (Erel)	-TOS: NW: 5.08; OB:8.10<br>-OSI: NW:2.85; OB:6.27<br>-Prolidase activity: NW: 58.33; OB: 68.96	-Prolidase activity was significantly related to plasma **TOS** levels (0.529, p<0.001) and **OSI** (0.519, p<0.001),<br>-BMI was significantly related to plasma **TOS** levels (0.721, p<0.001) and **OSI** (0.734, p<0.001),
Chen (2015)	China	379	Asia-Pacific region criteria<br><br>NW: BMI <23.0 kg/m2<br><br>Obesity: BMI ≥ 25 kg/m2	Colorimetric methods using (Nanjing Jiancheng Bioengineeri ng Institute, China)Chen (2015) does not specify which kit they used	-SOD: NW: 78.70; OB: 74.58<br>-MDA: NW:3.36; OB:3.69<br>-GPx: NW: 877.40; OB: 860.11<br>-Visfatin: NW: 41.24; OB:43.93	No correlations were made between the parameters: SOD, **MDA**, GPx, and visfatin.
Chrysohoou (2007)	Greece	310	WHO criteria<br><br>NW: BMI <25.0 kg/m2<br><br>Obesity: BMI ≥ 30	Colorimetric test in serum (ImAnOx, Immunodiag nostik AG, Ben- sheim, Germany).	-no other parameter	No correlations were made

			kg/m2			
Dambal (2011)	India	674	NW: BMI <25.0 kg/m2<br><br>Obesity: BMI ≥ 30 kg/m2	Phosphomolybdenum method	-SOD: NW: 2.26; OB: 3.22<br>-VitC: NW:0.23; OB: 0.12	No correlations were made
Dursun (2016)	Turkey	875	WHO criteria<br><br>NW: BMI <25.0 kg/m2<br><br>Obesity: BMI ≥ 30 kg/m2	Colorimetric using Fenton-type reaction 2,29-azino-bis (3-ethylbenz-thiazoline-6-sulfonic acid) radical cation by antioxidants (Erel)	-TOS: NW: 4.35; OB: 5.47<br>-OSI: NW: 2.58; OB: 3.49	**TOS** serum was significantly related to triglycerides (0.387, $p<0.05$)
Eren (2014)	Turkey	761	Expert Comittee<br><br>NW: healthy age- and gender matched participants<br><br>Obesity: BMI values over the 95th percentile for	Colorimetric using Fenton-type reaction 2,29-azino-bis (3-ethylbenz-thiazoline-6-sulfonic acid) radical cation by antioxidants	-Paraoxonase: NW:95.86;OB: 126.88<br>-Arylesterase: NW:83.04;OB: 101.34<br>-TOS: NW:18.89;OB:24.16<br>-OSI: NW: 1.90; OB: 2.21	No correlations were made between the parameters: paraoxonase, arylesterase, **TOS** and **OSI**

			age and gender	(Erel)		
Faienza (2012)	Italy	6	Italian growth charts<br><br>NW: BMI <1.7 SD<br><br>Obesity: BMI >2 SD for the mean for age and sex	BAP test spectrophotometric method (Diacron International)	-Oxidant status: d-ROMS test: NW:357; OB: 431.25<br>-BAP/d-ROMs ratio: NW: 6.85; OB: 5.06	-**d-ROMS** leves was significantly related to BMI SDS (0.14, p<0.0005) -**BAD/ROMs ratio** was significantly related to BMI SDS (0.2, p<0.0001), waist circumference (0.1), total cholesterol (0.06) and triglycerides (0.04).
García-Sánchez (2020)	Mexico	1.550	WHO criteria<br><br>NW: BMI <25.0 kg/m2<br><br>Obesity: BMI ≥ 30 kg/m2	Total Antioxidant Power Kit, No. TA02.090130, Oxford Biomedical Research	-LPO: NW: 4.30; OB: 3.80<br>-8-isoprostane: NW: 24.25; OB: 28.44<br>-Nitric Oxide: NW: 234.81; OB: 242.36<br>-SOD: NW: 0.50; OB: 0.34<br>-GPx: NW: 1.28; OB: 2.17<br>-CAT: NW: 18.07; OB: 18.35	-8-isoprostan**e** was significantly related to visceral abdominal fat(0.249, p<0.05) and BMI (0.261, p<0.05).<br>-LPO was significantly related to wait-hip index (0.274, p<0.05) and visceral abdominal fat (0.274, p<0.05)
Hadzovicc-Dzuvo (2015)	Herzegovina and Bosnia	1.283	NW: BMI 19-25 kg/m2<br><br>Obesity: BMI ≥ 30 kg/m2	Quantitative colorimetric assay, using Total antioxidant Capacity - QuantiCrom Antioxidant Assay Kit (BioAssay systems, USA; DTAC-100)	-no other parameter	No correlations were made

Karaozouzene (2011)	Algeria	678	NW: BMI 18-24 kg/m2<br><br>Obesity: BMI ≥ 30 kg/m2	Oxygen radical absorbance capacity of plasma (ORAC)	-VIT A: NW: 14.01; OB: 12.94<br>-VIT C: NW: 40.52; OB: 29.36<br>-VIT E: NW: 22.53; OB: 15.55<br>-CAT: data in graphs only<br>-GPx: data in graphs only<br>-SOD: data in graphs only<br>-Hydroperoxides: NW: 2.22; OB: 4.05<br>-Carbonyl proteins: NW: 3.41; OB: 5.64	-In the younger group BMI was significantly related to SOD (0.431, p<0.001), carbonyl proteins (0.372, p<0.01) and hydroperoxides (0.328, p<0.01).<br>-In the older group BMI was significantly related to carbonyl proteins (0.572, p<0.001), hydroperoxides (0.563, p<0.001) and SOD (-0.522, p<0.001)<br>-VitA, VitC, VitE, CAT, GPx were not significantly related to metabolic nor anthropometric parameters
Lejawa (2021)	Poland		<u>WHO criteria</u><br><br>NW: BMI <25.0 kg/m2<br><br>Obesity: BMI ≥ 30 kg/m2	Colorimetric assay using 2, 20-Azino-di-[3-ethylbenzthiazoline sulphonate] (ABTS) (Erel)	-TOS: NW: 4.05; OB: 4.57<br>-OSI: NW: 24; OB: 22<br>-MDA: NW: 1.40; OB: 1.48<br>-LHPs: NW: 1.71; OB: 2.8<br>-PSHs: NW: 4.63; OB: 4.64<br>-Ceruloplasmin: NW: 34.21; OB: 35.87<br>-SOD: NW: 19.54; OB: 18.95<br>-CuZn-SOD: NW: 8.72; OB: 8.23	-BMI was significantly related to **TOS** (0.27, p<0.05) and LPH (0.64, p<0.0001).<br>-Waist-to-Hip Ratio was significantly related to **ceruloplasmin** (0.24, p<0.05), LPH (0.39, p<0.001), CuZnSOD (-0.22, p<0.05) and **MDA** (0.25, p<0.05).<br>-Visceral adipose index was significantly related to **TOS** (0.43, p<0.0001), **OSI** (-0.26, p<0.05) and LPH (0.61, p<0.0001).<br>-Systolic blood pressure was significantly related with LPH (0.32, p<0.01)<br>Diastolic blood pressure was significantly related to ceruloplasmin (0.26, p<0.05.<br>-Total cholesterol was significantly related to **TOS** (0.24, p<0.05) and LPH (0.35, p<0.001).<br>-apoA1 was significantly related to LPH (-0.22, p<0.05)<br>-apoB was significantly related to **ceruloplasmin** (0.22, p<0.05), **TOS** (0.24, p<0.05) and LPH (0.41, p<0.0001).<br>-HDL-C was significantly related to **TOS** (0.31, p<0.01) and LPH (-0.51, p<0.0001).<br>-HDL % was significantly related to **TOS** (-0.22, p<0.01), and LPH (-0.55, p<0.0001)<br>-Triglycerides was significantly related to **TOS** (0.50, p<0.0001), **OSI** (-0.32, p<0.01) and LPH (0.65, p<0.0001)<br>-hsCRP was significantly related to ceruloplasmin (0.34, p<0.001), **TOS** (0.32, p<0.001) and LPH (0.40, p<0.0001)<br>-Glucose was significantly related to PSH (0.21, p<0.05), **TOS** (0.24,

						p<0.05), and LPH (0.24, p<0.05)<br>-HbA1c was significantly related to ceruloplasmin (0.09, p<0.05) and **TOS** (0.27, p<0.05),<br>-Uric acid was significantly related to ceruloplasmin (0.22, p<0.05), **TOS** (0.28, p<0.05) and LPH (0.46, p<0.0001)<br>-GGT was significantly related to ceruloplasmin (0.36, p<0.001)<br>-ALP was significantly related to PSH (0.24, p<0.05), **TOS** (0.24, p<0.05) and LPH (0.23, p<0.05)<br>-ALT was significantly related to ceruloplasmin (0.21, p<0.05), and LPH (0.42, p<0.0001).<br>-AST was significantly related to ceruloplasmin (0.26, p<0.05) and LPH (0.30, p<0.01)<br>-LDH was significantly related to ceruloplasmin (0.22, p<0.05), **TOS** (0.23, p<0.05), LPH (0.50, p<0.0001) and CuZnSOD (-0.26, p<0.05)
Mahasneh (2016)	USA		NW: BMI <25.0 kg/m2<br><br>Obesity: BMI ≥ 30 kg/m2	AOP-490 kit (Oxis Interna-tional, Foster City, CA).	-TGSH: NW: 43.3; OB: 39.1<br>-CAT: NW: 162.3; OB: 156.3<br>-GR: NW: 32; OB: 35.4<br>-GPx: NW: 101.3; OB: 83.8<br>-CuZn-SOD: NW: 29.8; OB: 31.1<br>-MPO: NW: 29.7; OB: 42.9	No correlations were made
Matusik (2015)	Poland	299	<u>Who charts</u><br><br>NW:<br><br>Obesity: BMI above the 95th percentile for age and sex	Photometric technique (ImmunDiag nostik, Bensheim, Germany).	TOS: NW: 418.41; OB: 420.08<br>GPx: NW: 83.98; OB: 72.66	**TOS** was significantly related to BMI Z- score (SD) (0.245, p<0.05), Waist-to-hip ratio (0.273, p<0.05), fat mass (0.423, p<0.0001), fat free mass (-0.423, p<0.0001), total body water (-0.423, p<0.0001) and predicted muscle mass (-0.419, p<0.01),<br>-GPx was not significantly related to metabolic nor anthropometric parameters.
Park (2016)	Korea	301	<u>BMI criteria for Asian</u><br><br>NW: BMI <25	Commercial TAS kit (Randox Laboratories Ltd, London,	-TBARS: NW: 6.30; OB: 6.94	No correlations were made

			kg/m2<br><br>Obesity: BMI >25 kg/m2	UK).		
Pirgon (2013)	Turkey	1.023	NW:<br>Obesity: BMI of ≥95th percentile according to reference curves for Turkish	Colorimetric assay using 2, 20-Azino-di-[3-ethylbenzthiazoline sulphonate] (ABTS) (Erel)	-TOS: NW: 5.85; OB: 8.68<br>-OSI: NW: 7.36; OB: 13.93	-**TOS** and **OSI** were not significantly related to metabolic nor anthropometric parameters
Rowicka (2017)	Polonia	86	Who charts<br><br>NW:<br><br>Obesity: BMI above the 95th percentile for age and sex	Colorimetric assay (Labor Diagnostica Nord GmbH & Co. KG, Nordhorn, Germany).	-TOS: NW: 0.15; OB: 0.22<br>-OSI: NW: 0.11; OB: 0.18	-**TOS** was significantly related to duration and obesity (0.32, p<0.01).<br>**-OSI** was not significantly related to metabolic nor anthropometric parameters.
Skalicky (2008)	Czech Republic	218	WHO criteria<br><br>NW: BMI <25.0 kg/m2<br><br>Obesity: BMI ≥ 30 kg/m2	Kit (Randox, Crumlin, UK).	-Free radicals: NW: 4.71; OB: 7.51<br>-MDA: NW: 1.18; OB: 2.56<br>-Alfa Antiproteinase: NW: 26.59; OB: 37.09<br>-GSSG/GSH: NW: 6.53; OB: 13.69<br>-VIT E: NW: 23.22; OB: 22.94<br>-Ceruloplasmin: NW: 0.38; OB: 0.22	-The values of the correlations are not mentioned.
Sonoli (2015)	India	801	Who criteria for the asian adults<br><br>NW: BMI <23	Korocevic et.al method by a Fenton type reaction	-TBARS: NW: 4.69; OB: 6.77<br>-Ceruloplasmin: NW:78.33; OB: 96.31	-In females waist-to-hip ratio was significantly related to ceruloplasmin (0.394, p<0.01) and TBARS (0.478, p<0.01)<br>-In males waist-to-hip ratio was significantly related to ceruloplasmin (0.532, p<0.01). TBARS was not significantly related to metabolic nor anthropometric parameters

			kg/m2<br><br>Obesity: BMI >28 kg/m2			
Vehapoglu (2016)	Turkey	27	NW: BMI in the 18.5th to 95th percentile<br><br>Obesity: BMI >95th percentile	Colorimetric using Fenton-type reaction 2,29-azino-bis (3-ethylbenz-thiazoline-6-sulfonic acid) radical cation by antioxidants (Erel)	-TOS: NW: 10.27; OB: 11.33<br>-OSI: NW: 0.81; OB: 0.96<br>-Total thiol: NW: 0.36; OB: 0.33	-**OSI** was significantly related to HOMA-IR (0.157, $p<0.05$) and insulin (0.154, $p<0.05$)
Aysegül (2014)	Turkey	27	International Task Force of Obesity in Child- hood and population-specific data<br><br>Obesity: BMI >95th percentile	Colorimetric assay using 2, 20-Azino-di-[3-ethylbenzthi azoline sulphonate] (ABTS) (Erel)	-TOS: NW: 5.25; OB: 6.08<br>-OSI: NW: 1.92; OB: 2.65	-**TOS** and **OSI** were not significantly related to metabolic nor anthropometric parameters

Supplementary Table S3. Reason of exclusion

Author	Year	Reason of exclusion
Abbasihormozi	2016	TAC was measured in seminal fluid
Alipour	2020	All patients included had type 2 diabetes
Besagil	2022	The mix of overweight subjects in the NW
Choi	2011	The mix of overweight subjects in the SO group
Colak	2020	The case group included subjects with overweight and obesity
Delvarianzadeh	2017	No comparison of SO *versus* NW, instead metabolic syndrome versus non-metabolic syndrome
Di Segni	2017	Studies performed in patients with polycystic ovary syndrome
Fariba	2015	All patients included had type 2 diabetes
Gulcan	2013	No comparison of SO *versus* NW, instead metabolic syndrome versus non-metabolic syndrome
Jakubiak	2021	The data only were shown in plots
Jakubiak	2022	No comparison of SO *versus* NW, instead metabolic syndrome versus non-metabolic syndrome

Karamouzis	2011	No comparison of SO *versus* NW, instead metabolic syndrome versus non-metabolic syndrome
Khalili	2022	All patients included had type 2 diabetes
Leite Almeida	2021	The case group included subjects with overweight and obesity
Lewandovski	2020	The mix of overweight subjects in the NW
Lim	2012	The mix of overweight subjects in the NW
Limberg	2014	The sample size was lower than 20 by group and Newcastle-Ottawa score was 5.
Mancini	2008	The mix of overweight subjects in the NW
Melissas	2006	The sample size was lower than 20 by group
Mizgier	2021	Studies performed in patients with polycystic ovary syndrome
Mokhtary	2022	All patients included had type 2 diabetes
Mohieldin	2014	The study groups were prediabetes versus normal fasting glycemia; thus, patients were not classified in obese versus normal weight subjects
Molnár	2004	The sample size was lower than 20 by group
Pilch	2021	The sample size was lower than 20 by group
Ratajczak	2020	The sample size was lower than 20 by group

Skalicky	2009	Duplicated publication
Söylemez	2010	Article written in Turkish
Valezi	2011	Low score on the Newcastle-Ottawa scale
Veigas	2011	The case group included subjects with overweight and obesity
Villalva	2015	The case group included subjects with overweight and obesity

TAC: Total Antioxidant Capacity, WHO: World Health Organization.

Supplementary Table S4. General characteristics of the included studies.

Author, year	Country	Inclusion criteria	Exclusion criteria	n obese, n controls, n total	Age at inclusion by group	Mean BMI by group	Proportion of Male: Female	Intra and inter-assay CV for TAC method of quantification

Amirkhizi (2010)	Iran	Women aged 20-45 years of any BMI selected by a multiple cluster random sampling method that received attention at rural health centers of Kerman Province, Iran.	Pregnant, lactating and smoker women and participants with a prior history of cancer, cardiovascular disease, diabetes, renal or liver diseases, and those taking vitamin or mineral supplements.	Obese: 25<br>Control: 79<br>Total: 104	Obese: 39<br>NW: 27	Obese: 33.6<br>NW: 23.4	Male: 0<br>Female: 1	Intra: not exceed 4%<br><br>Inter: not exceed 7%
Asghari (2021)	Iran	Aged between 20 and 60 years old, and BMI 30 kg/m2 for subjects with obesity and BMI <25 for healthy normal weight controls.	Pregnancy, breastfeeding, consumption of any antioxidant supplements within the previous three months, being on weight loss diets for at least three months prior to participation in the study, receiving cholesterol-lowering medication, estrogen, progesterone, or diuretics; the history of acute or chronic liver diseases, kidney dysfunctions, diabetes, and other endocrine disorders, rheumatoid arthritis, cardiovascular diseases, thyroid disorders,	Obese: 140<br>Control: 90<br>Total: 230	Obese: 41<br>NW: 40.2	Obese: 32.3<br>NW: 23.6	Male: 0.58<br>Female: 0.42	-

			autoimmune or endocrine disorders, and chemotherapy during the previous year.					
Aslan (2017)	Turkey	BMI was calculated by dividing weight by height (kg/m2).<br>The healthy control groups were constituted with healthy age-and gender matched asymptomatic participants who had normal physical examination and clear medical history, were not receiving any drugs, and were not smoking or consuming alcohol and had no known systemic disease.	The obese subjects with those circumstances were excluded from study: diabetes mellitus, hyperlipidemia, chronic hypertension disease, coronary artery disease and hepatic or renal disease as well as usage of supplemental vitamins and smoking.	Obese: 27<br>Control: 26<br>Total: 53	Obese: 30<br>NW: 28	Obese: 36.31<br>NW: 21.5	Male: 0.58<br>Female: 0.42	-
Chen (2014)	China	Obese men range 40 to 47, normal-weight men range 41 to 47. The BMI cut-off points were respectively and, according to obesity diagnosis standards in the Asia-Pacific region and was calculated as the ratio between weight and height squared (in kg/m2).	History of high blood pressure, diabetes, cardiovascular disease, stroke, peripheral vascular disease, and disorders of the liver and kidney. Subjects who were taking drugs including medica- tions known to reduce lipid, such as statins, or affect metabolism.	Obese: 31<br>Control: 30<br>Total: 61	Obese: 44.9<br>NW: 44.03	Obese: 28.9<br>NW: 22.7	Male: 1<br>Female: 0	-
Chrysohoou (2007)	Greece	Habitants from the province of Attica,	Chronic viral infections, colds or flu,	Obese: 540	Obese: 50<br>NW: 41	Obese:<br>NW:	Male: 0.40<br>Female: 0.60	Intra: not exceed 3%

		aged from 18 to 89 years of age, and without any clinical evidence of cardiovascular disease, were randomly selected. Overweight was defined as BMI between 25 and 29.9 kg/m2, while obesity as BMI greater than 29.9 kg/m2 based on the WHO criteria.	acute respiratory infections, dental problems or any type of surgery in the week preceding the study.	Control: 1226<br>Total: 1766				Inter: not exceed 3%
Dambal (2011)	India	Obesity status was categorized by the BMI. The study was carried out on five groups of subjects in the age group of 40-60 years. Fifty non-diabetic and non-obese formed the Control group and the study group formed by Fifty obese individuals (BMI 30kg/m2 and above)	-	Obese: 50<br>Control: 50<br>Total: 100	Obese:<br>NW:	Obese:<br>NW:	Male: 0.40<br>Female: 0.60	-
Dursun (2016)	Turkey	Obese women women who presented to the Endocrinology Outpatient Clinic at Hacettepe University consecutively and age, sex-matched lean controls. Used (WHO)-defined BMI-based categories of underweight (BMI	Systemic disease, periodontal treatment history, antibiotics, anti- inflammatory, or any other drug use during the past 3 months, being smoker and use of alcohol or antioxidant vitamin tablet.	Obese: 40<br>Control: 20<br>Total: 20	Obese: 27.29<br>NW: 26.11	Obese: 34.08<br>NW: 22.15	Male: 0<br>Female: 1	-

		<18.5kg/m2), normal weight (BMI1⁄418.5–24.9kg/m2), overweight (BMI1⁄425.0–29.9kg/m2), and obese (BMI30kg/m2) to determine the study groups as lean (normal weight [BMI1⁄418.5–24.9kg/ m2]) and obese (BMI 30 kg/m2).						
Eren (2014)	Turkey	Body mass index values over the 95th percentile for age and gender were accepted as obese. Obese cases were divided into two groups as those with MetS and those without MetS. Patients with MetS were defined as subjects showing three or more of the following parameters: abdominal obesity (increased waist circumference, increased waist-height ratio), hypertension, hyperglycemia-insulin resistance, high triglyceride (TG) levels, low HDL levels. The control group was composed of healthy age and gender matched participants.	Have systemic diseases and syndromic diseases.	Obese: 95<br>Control: 56<br>Total: 151	Obese: 13.34<br>NW: 13.95	Obese: 32.14<br>NW: 18.85	Male: 0.43<br>Female: 0.57	-

Faienza (2012)	Italy	All the obese subjects were affected by severe obesity (BMI >2 SD for the mean for age and sex). Twenty-five of them had MetS and the remaining 30 had simple obesity. Obesity was defined in presence of BMI greater than 95th percentile for age and sex, in accordance with Italian growth charts. As control group, normal-weight children BMI <1.7 SD), who had been admitted to our Department of Pediatrics for minor diseases.	Obese group: endocrinological disorders or genetic syndromes associated with obesity, acute infection or presence of diseases that could have an influence on oxidative stress (i.e. asthma), subjects under some form of medication (i.e. antioxidant vita- mins such as ascorbate, tocopherols, alphacarotene, or polyphenol-containing nutraceuticals) during or a week before the blood samples were taken.<br><br>Control group: hypertension, dyslipidemia and acute infections.	Obese: 55<br>Control: 30<br>Total: 85	Obese: 11.4<br>NW: 10.4	Obese: 2.22<br>NW: 0.37	Male: 0.51<br>Female: 0.49	-
García-Sánchez (2020)	Mexico	The study subjects were divided according to the criteria of the World Health Organization (WHO) for BMI in normal weight (18.5–24.9 kg/m2), overweight (25–29.9 kg/m2) and obesity (≥30 kg/m2).	Subjects that reported cerebrovascular disease, hepatitis, kidney disease, or those who were taking antioxidants.	Obese: 33<br>Control: 23<br>Total: 56	Obese: 56.45<br>NW: 68.7	-	Male: 0.25<br>Female: 0.75	Intra: 5.70%<br><br>Inter: -

Hadzovicc-Dzuvo (2015)	Herzegovina and Bosnia	Postmenopausal women with preserved bone mass determined by their total hip and/or total lumbar bone mineral density measured by densitometry. BMI values in the range 19-25 kg/m2 were considered normal weight, while ≥30 kg/m2 were the cut-off levels for obese subjects.	Receiving hormone replacement therapy or had received it within the last year before the start of the study or had cancer, cardiovascular disease, diabetes mellitus and other endocrinal disorders, bronchial asthma, acute or chronic inflammatory diseases, autoimmune diseases and rheumatic diseases.	Obese: 23<br>Control: 36<br>Total: 59	Obese:<br>NW:	Obese:<br>NW:	Male: 0<br>Female: 1	-
Karaouzene (2011)	Algeria	Men who attended annual general health examinations were recruited from the department of endocrinology, University Hospital of Tlemcen (Algeria), with primary criteria including BMI 18e24 Kg/m2 or 30 Kg/m2, age range between 20 and 75 years.	Taking any medication known to influence lipid metabolism or vitamin supplements and having chronic diseases.	Obese: 85<br>Control: 120<br>Total: 205	Obese: 48<br>NW: 46	Obese: 33.2<br>NW: 23.5	Male: 1<br>Female: 0	-
Lejawa (2021)	Poland	Healthy slim individuals without MetS and 49 obese patients matched by age divided into three subgroups according to body mass index (kg/m2) and metabolic health: metabolically healthy normal weight metabolically healthy obese and	-	Obese: 49<br>Control: 49<br>Total: 98	Obese: 30.5<br>NW: 30.9	Obese: 32.6<br>NW: 23.36	Male: 1<br>Female: 0	-

		metabolically unhealthy obese. Obesity defined according to the WHO classification as BMI ≥ 30 kg/m2. Metabolic health was defined according to the 2009 International Diabetes Federation.						
Mahasneh (2016)	USA	Women who had provided a blood sample to DBBR between April 1, 2004 and August 31, 2007, and had complete questionnaire data on all variables of interest.	No personal history of cancer.	Obese: 35<br>Control: 46<br>Total: 81	-	-	Male: 0<br>Female: 1	-
Matusik (2015)	Poland	Obesity was defined as BMI at or above the 95th percentile for age and sex, using the WHO charts<br><br>Control group: all healthy, normal weight, and did not use neither medications nor diet supplements.	Children with syndromic obesity and endocrine disorders associated with obesity, factors that could influence oxidative status like infections, chronic diseases (i.e., asthma), and medications (i.e., antioxidant vitamins).	Obese: 78<br>Control: 82<br>Total: 160	Obese: 13.96<br>NW: 13.72	Obese: 2.96<br>NW: 0.38	Male: 0.52<br>Female: 0.48	-
Park (2016)	Korea	Subjects were divided into the non-obese group and the obese group based on BMI criteria for Asians suggested by the	Current user of vitamin supplements, current drug-user for inflammatory disease (e.g., Crohn disease, rheumatoid arthritis),	Obese: 33<br>Control: 45<br>Total: 78	Obese: 65.3<br>NW: 66.6	Obese: 26.3<br>NW: 22.5	Male: 0.55<br>Female: 0.45	-

		International Obesity Task Force: BMI <25 kg/m2 (non-obese) and BMI ≥ 25 kg/m2 (obese).	dyslipidemia, or hypertension, or concurrent or recent participant in another intervention study.					
Pirgon (2013)	Turkey	Obese adolescents age range 12-17 years, were randomly recruited, None of the patients had a family history for type 2 diabetes. Patients with a BMI of ≥95th percentile according to reference curves for Turkish children and adolescents were accepted as obese<br><br>Lean adolescents as a control group consisted of healthy adolescents who attended the hospital for minor illnesses such as common cold, conjunctivitis, or other similar conditions. None of the subjects were vegetarian.	Have hepatic virus infections (hepatitis A, B, C, cytomegalovirus and Epstein-Barr virus infections), alcohol consumption, history of parenteral nutrition, and use of drugs known to induce steatosis (e.g. amiodarone, glucocorticoids, L-asparaginase, valproic acid) or to affect body weight and carbohydrate metabolism. Autoimmune and metabolic liver disease, Wilson's disease, and α-1-antitrypsin- associated liver disease were ruled out using standard clinical and laboratory criteria.	Obese: 46<br>Control: 29<br>Total: 75	Obese: 12.5<br>NW: 12.7	Obese: 30.63<br>NW: 18.36	Male: 0.48<br>Female: 0.52	-
Rowicka (2017)	Polonia	Children with obesity, wherein the criterion for obesity diagnosis in children up to 5 years old was BMI z-score ≥ 3SD, and in children over 5 years old BMI z-score ≥ 2SD.	Infections of various etiologies and localizations as well as intake of prescription medications and food supplements with antioxidant properties.	Obese: 62<br>Control: 21<br>Total: 83	Obese: 7.5<br>NW: 6.4	Obese: 23.5<br>NW: 19.5	Male: 0.39<br>Female: 0.61	Intra: 2.5%<br><br>Inter: 3.33%

		Nonobese children whose BMI z-score was <−1 + 1>.						
Skalicky (2018)	Czech Republic	obese adults characterized by waist circumference, body mass index (BMI) and age. They was divided into two sub- groups patients fulfilling at least three criteria of MetS and without expressed MetS symptoms.The control group consisted of healthy normal-weight.	Had any serious health complications. Renal, hepatic, gastrointestinal, pulmonary, endocrine or oncological diseases.	Obese: 40<br>Control: 48<br>Total: 88	Obese: 50<br>NW: 52.12	Obese: 35.3<br>NW: 21.86	Male: 0.52<br>Female: 0.48	-
Sonoli (2015)	India	BMI ≤23 were considered as normal and BMI ≥28 were considered as obese, as per the WHO criteria for the Asian adults.	Overweight individuals, pregnant and menstruating women during the time of blood collection, and individuals suffering from acute, chronic diseases, and on any type of treatment for obesity.	Obese: 70<br>Control: 35<br>Total: 105	Obese: 23.1<br>NW: 22.9	Obese: 31.38<br>NW: 23.07	Male: 0.5<br>Female: 0.5	-
Vehapoglu (2016)	Turkey	Prepubescent children aged 2–11 years, obese children (with a BMI ≥the 95th percen- tile), and 80 healthy children of normal- weight (with a BMI in the 18.5th to 95th percentile). Dietary history with adequate nutrient intake (in quality and quantity).	Endocrine disease (Cushing's syndrome or hypothyroidism), acute or chronic inflammatory disease, malabsorption syndromes such as celiac disease or cystic fibrosis, infections or systemic illnesses, and the use of prescription medications, vitamins,	Obese: 90<br>Control: 80<br>Total: 170	Obese: 7.4<br>NW: 7.2	Obese: 25.78<br>NW: 16.75	Male: 0.47<br>Female: 0.53	Intra: 2.38%<br><br>Inter: 4%

			or mineral supplements for any reason.					
Aysegül (2014)	Turkey	Obesity was defined as a body mass index (BMI) > 95th percentile using the definition of the International Task Force of Obesity in Child- hood and population-specific data.<br><br>Control group was recruited among the healthy children who were admitted to the pediatric clinics for routine yearly examination.	Syndrome (Prader Willi, Laurence-Moon-Biedle syndrome, etc.) and endocrine causes (Cushing's Syndrome, hypothyroidism, etc.) of obesity. Use of medications or had a history or evidence of current metabolic, cardiovascular, respiratory or hepatic disease. Patients under vitamin and/or mineral supplements.	Obese: 38<br>Control: 51<br>Total: 89	Obese: 9.42<br>NW: 9.29	Obese: 27.63<br>NW: 17.42	Male: 0.52<br>Female: 0.48	-

Supplementary Table S5. Correlations of co-determined redox parameters with anthropo-metabolic biomarkers

Parameter	Number of studies estimating correlations with anthropo-metabolic parameters	Correlations reported

**MDA**	Two<br>1. Amirkhizi (2010)<br>2. Lejawa (2021)	**Study 1**. Plasma MDA was significantly co-related to weight (r=0.185, p<0.01), BMI (r=0.484, p<0.0001), waist circumference (r=0.582, p<0.0001), and waist-to-hip ratio (r=0.474, p<0.0001).<br>**Study 2**. Plasma MDA was significantly co-related to Waist-to-Hip Ratio (r=0.474, p<0.0001).

**TOS**	Eight	**Two** studies. No reported correlations of TOS with anthropo-metabolic parameters.<br>**Two** studies. Reported no significant correlations of TOS with anthropo-metabolic parameters.<br>F**ive** studies reported significant correlations with:<br>-BMI (0.721, p<0.001), (0.27, p<0.05), and (0.245, p<0.05)<br>-Triglycerides (0.387, p<0.05) and (0.50, p<0.0001)<br>- Visceral adipose index (0.43, p<0.0001)<br>-Total cholesterol (0.24, p<0.05)<br>-ApoB (0.24, p<0.05)<br>-HDL-c (0.31, p<0.01)<br>-hsCRP (0.32, p<0.001)<br>-Glucose (0.24, p<0.05)<br>-HbA1c (0.27, p<0.05)<br>-Uric acid (0.28, p<0.05)<br>-ALP (0.24, p<0.05) and<br>-LDH (0.23, p<0.05)<br>-Waist-to-hip ratio (0.273, p<0.05)<br>-Fat mass (0.423, p<0.0001)<br>-Fat free mass (-0.423, p<0.0001)<br>-Predicted muscle mass (-0.419, p<0.01)<br>-Obesity duration (0.32, p<0.01)

**OSi**	Five	**Three** studies reported significant correlations of OSi with:<br>-BMI (0.734, p<0.001)<br>-Visceral adipose index (-0.26, p<0.05)<br>-Triglycerides (-0.32, p<0.01)<br>-HOMA-IR (0.157, p<0.05)<br>-Insulin (0.154, p<0.05)<br>**Two** studies reported no significant correlations of OSi with anthropo-metabolic parameters.

MDA: malondialdehyde; TOS: Total oxidant status; OSi: Oxidative stress index.

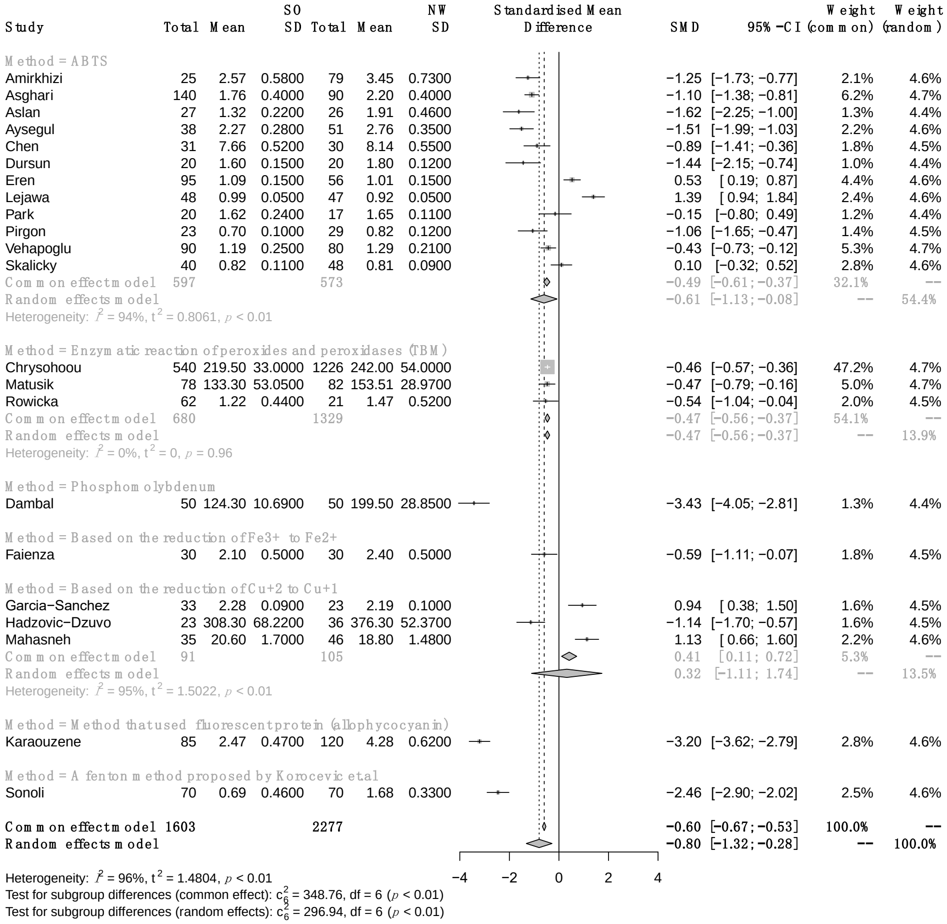

Supplementary Figure S1. Forest plot exhibiting a subgroup analysis on the difference in TAC between SO and NW by TAC measurement method. SD, standard deviation; SMD, standardized mean difference; CI, confidential interval.

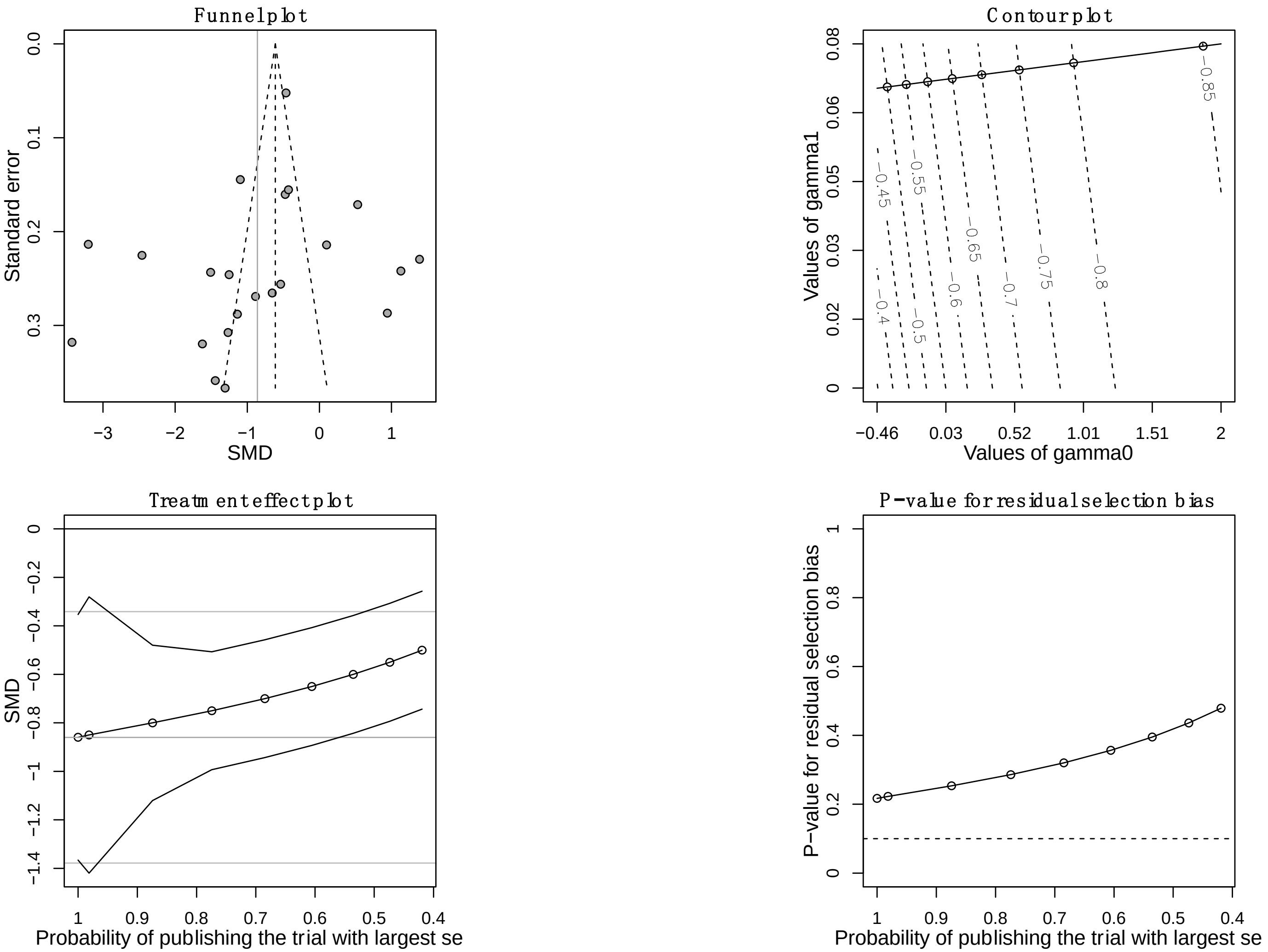
Funnel plot
Standard error
SMD
Contour plot
Values of gamma1
Values of gamma0
Treatment effect plot
SMD
Probability of publishing the trial with largest se
P−value for residual selection bias
P−value for residual selection bias
Probability of publishing the trial with largest se

Supplementary Figure S2. Publication bias and systematic heterogeneity represented through funnel plot and COPAS method.
